# Evidence that nonsignificant results are sometimes preferred: Reverse *P*-hacking or selective reporting?

**DOI:** 10.1371/journal.pbio.3000127

**Published:** 2019-01-25

**Authors:** Pierre J. C. Chuard, Milan Vrtílek, Megan L. Head, Michael D. Jennions

**Affiliations:** 1 Division of Ecology and Evolution, Research School of Biology, The Australian National University, Canberra, Australia; 2 Department of Biological Sciences, Bishop's University, Sherbrooke, Canada

## Abstract

There is increased concern about poor scientific practices arising from an excessive focus on *P*-values. Two particularly worrisome practices are selective reporting of significant results and ‘*P*-hacking’. The latter is the manipulation of data collection, usage, or analyses to obtain statistically significant outcomes. Here, we introduce the novel, to our knowledge, concepts of selective reporting of nonsignificant results and ‘reverse *P*-hacking’ whereby researchers ensure that tests produce a nonsignificant result. We test whether these practices occur in experiments in which researchers randomly assign subjects to treatment and control groups to minimise differences in confounding variables that might affect the focal outcome. By chance alone, 5% of tests for a group difference in confounding variables should yield a significant result (*P* < 0.05). If researchers less often report significant findings and/or reverse *P*-hack to avoid significant outcomes that undermine the ethos that experimental and control groups only differ with respect to actively manipulated variables, we expect significant results from tests for group differences to be under-represented in the literature. We surveyed the behavioural ecology literature and found significantly more nonsignificant *P*-values reported for tests of group differences in potentially confounding variables than the expected 95% (*P* = 0.005; *N* = 250 studies). This novel, to our knowledge, publication bias could result from selective reporting of nonsignificant results and/or from reverse *P*-hacking. We encourage others to test for a bias toward publishing nonsignificant results in the equivalent context in their own research discipline.

## Abstract 2

There is concern that the scientific literature is biased towards reporting statistically significant results. By contrast, this Perspective article presents evidence for an unusual situation in which there is a systematic bias towards publishing non-significant findings.

## Introduction

The publication record of academics is a key factor determining funding and career success [1]. This constant pressure to publish, and to do so in high-impact journals that strongly favour significant results [2], has consequences for the extent to which published scientific studies reflect the outcome of all original research. Key studies in many fields are not replicated [3], and if they are, the original results are often not reproduced [4,5]. The latter phenomenon is widely known as the replication crisis [5,6]. One reason posited for low replicability is the existence of a publication bias that favours reporting significant findings and suppressing nonsignificant ones [7].

**Fig 1:**
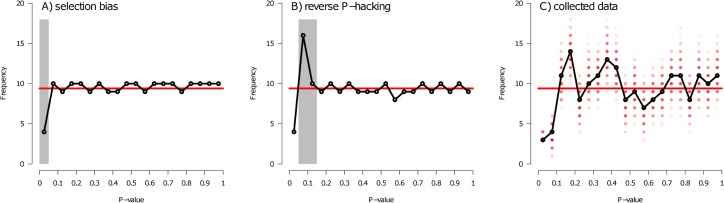
Frequency distribution of numerical *P*-values (*P*-curve). Two hypothetical examples of *P*-curves show the expected distribution if (a) selection bias is present or (b) certain types of reverse *P*-hacking occur (see [12]). Data collected from articles testing the effect of confounding variables between treatment(s) and control groups are shown in (c). In (c), darker red points represent a higher frequency of *P*-values/bin among the randomised datasets. The horizontal red line denotes the theoretical even distribution across 20 bins (188 articles in 20 bins = 9.4 articles/bin). The grey rectangles emphasize intervals of interest in the two hypothetical scenarios. We only used *P*-values presented to at least two decimal places. The full data file of the 1,805 articles consulted and the 250 from which data were extracted and the *R* scripts used for the analysis and to generate the figures are available at https://figshare.com/s/f3bb7dfefdaa8976d3a1 or https://osf.io/au7yc/.

There are two main types of publication bias. First, selection bias occurs when studies either go unpublished (‘file-drawer effect’) or statistical tests are not reported within published studies (selective reporting) (Fig 1A). It often reflects a tendency by authors, reviewers, and editors, respectively, to preferentially submit, recommend, and accept studies with statistically significant findings (usually *P* < 0.05) for publication [2,8]. It results in artificially increased estimates of average effect sizes because some conducted studies with smaller effect sizes (i.e., larger *P*-values for a given sample size) are missing from the scientific literature and excluded from meta-analyses or synthetic reviews. Second, *P*-hacking occurs when effect sizes are inflated because of how data are collected or analysed (Fig 1B). It involves the active, although not necessarily conscious, manipulation of data, data collection, or statistical tests [9,10] to obtain a statistically significant result for a favoured explanatory variable. This can occur by, for example, stopping a study as soon as a significant result is obtained rather than collecting a prespecified sample size, removal of outliers, and inclusion or exclusion of terms from an initially chosen statistical model. Although there are methods to detect missing, unpublished results that arise because of selection bias (for example, the use of funnel plots of effect sizes that should be symmetric) [11], correcting for *P*-hacking is notoriously difficult. This is partly because the various types of *P*-hacking practices will have different effects on the distribution of *P*-values that result when a pool of studies are considered, some of which have been *P*-hacked [12].

Well-conducted experimental studies allow researchers to reduce the effect of confounding factors that might otherwise hinder the ability to attribute causal effects to manipulated variables. We define a confounding variable as an unmanipulated variable that might affect the interpretation of an experiment if it differs between control and treatment groups or among different treatment groups. For example, when studying the effect of manipulating testosterone levels on male mating success, male body size could be considered a confounding variable because larger males tend to be more attractive to females in many species [13]. There are two main approaches to minimise the effect of confounding variables in experiments: balanced designs [14] and randomisation [15]. In a balanced design, a key confounding variable is purposefully distributed across treatment groups to avoid differences in group means. This is usually done by sequentially assigning subjects to treatment groups based on their rank for the confounding variable (for example, from the largest to the smallest individual). Randomisation, on the other hand, is when subjects are randomly assigned to different treatment groups. Whichever approach investigators use, one might then expect them to test for differences among groups in the confounding variable to ensure and demonstrate that they have successfully eliminated it as a source of between-group variation in the focal outcome variable [16]. Of course, by chance alone, the difference among groups in a confounding variable can be sufficiently large that it is statistically significant. Given random assignment of subjects, the probability of significance is 5% if we use the typical significance level of 0.05 employed in biological studies [17]. The probability of significance for the main confounding variable is far smaller for experiments with a deliberately balanced design [14], although, of course, other confounding variables that are uncorrelated with the main confounding variable are still being randomly assigned. Randomisation is therefore less effective than a balanced design, but it is still the most common approach to assigning subjects to experiments in ecology [18,19] and is the gold standard in randomised control trials in medicine [17].

Significant among-group differences in confounding variables are seen to potentially undermine the conclusions of an experimental study [16,17]. Researchers might therefore be tempted not to report significant differences in confounding variables between treatment(s) and control(s) to avoid criticism from reviewers. This could lead to selective reporting of nonsignificance tests. Or researchers might manipulate data collection or analyses (for example, by including or removing some subjects from a dataset) until group differences in confounding variables become statistically nonsignificant. We refer to this type of active data manipulation as ‘reverse *P*-hacking’. Here, we are specifically interested in those forms of *P*-hacking that will lead to slight shifts in *P*-values with cumulative testing until the ‘desired’ threshold is exceeded (see [9,12]) (see Fig 1).

We test for the presence of selective reporting of nonsignificance and/or reverse *P*-hacking, using articles about behavioural ecology. We chose this discipline because it is the one with which we are most familiar, which facilitated identifying suitable papers. If researchers honestly report all conducted statistical tests for confounding variables between treatment and control groups after random assignment of subjects, we expect 5% to report a statistically significant difference.

## Assessing the extent of selective reporting of negative results and reverse *P*-hacking in the literature

We preregistered our study design (https://osf.io/xg6ja/), and subsequent modifications were explicitly noted. The full details of the database are provided in S1 Text. In brief, we extracted data from 250 of 1,805 articles examined from three top behavioural ecology journals (1990–2018) that reported on experimental studies. We recorded all *P*-values or statements about the significance of results from tests of whether the mean value of confounding variables differed between treatment(s) and control groups. To be conservative, we treated an article as ‘significant’ if it contained at least one significant outcome. We then tested the null hypothesis that 5% of articles that test for such a difference will report a significant *P*-value (i.e., *P* < 0.05) (Fig 1A). To investigate reverse *P*-hacking, we examined *P*-curves (Fig 1B) [10], using all *P*-values presented to two or more decimal places (188 articles, 427 *P*-values). We tested whether there are more *P*-values in the bin closest to the significance threshold (0.05 to < 0.10) than in the next bin (0.10 to < 0.15) (Fig 1B [9]; but see [12]). We iteratively performed the test by randomly drawing one *P*-value per article. We present the median *P*-value.

The 250 articles with usable data contained 510 tests. Of these articles, only four (1.6%) reported at least one significant difference between the treatment and control group in the mean value of a confounding variable. This is significantly fewer articles than the expected 5% (i.e., 12.5 articles) that should occur in the absence of publication bias (exact binomial test: CI = 0–0.036, *P* = 0.005). Furthermore, two of the four articles with significant test outcomes also had several nonsignificant test outcomes (one article had two tests, and the other had eight tests). If we repeatedly randomly sampled a single test per article, this leads to an even lower presence of significant findings (predominantly only two articles [0.8%] with significant tests, CI = 0–0.025, *P* < 0.001).

The number of *P*-values in the 0.05 to < 0.10 bin immediately above the significant threshold was not significantly larger than the number in the adjacent 0.10 to < 0.15 bin (exact binomial tests, median *P* = 0.989, standard deviation [SD] = 0.036, Fig 1C). The pattern predicted given the type of reverse *P*-hacking we were testing for (see [9,12]) was therefore not seen (see prediction in Fig 1A). Instead, in addition to there being significantly fewer than expected *P*-values below 0.05 (see Methods for analysis below), there were equally few *P*-values in the 0.05 to 0.10 bin (Fig 1C). Based on this pattern (see Fig 1B), there therefore appears to be selective reporting. However, because there are fewer than expected studies with *P*-values below 0.10 rather than 0.05, the pattern suggests a bias against reporting *P*-values below 0.10.

There was no difference in the year of publication between articles that did or did not report at least one significant difference between the treatment and control group in the mean value of a confounding variable (independent sample *t* test: *t*_247_ = 0.408, *P* = 0.342; significant: year 2004.5 ± 3.1, nonsignificant: year 2005.9 ± 0.5; mean ± SE, *n* = 4, 246). This test has little statistical power (15%) to detect a medium-strength effect (*d* = 0.5) [20], but we include it because it was part of our preregistered study design.

Failure to identify a confounding variable can be a serious flaw if it has a strong causal effect on the focal outcome of an experiment (but see [17]). Depending on the direction of a difference in the confounding variable between the control and treatment group, it could either inflate or obscure the true effect of an experimentally manipulated variable. This is most likely to be an issue when the confounding variable is strongly related to the measured outcome of an experiment (for example, body size is correlated with life span in dogs). We compiled *P*-values (or statements about significance) from statistical tests of differences between treatment and control groups in the mean value of confounding variables. Given no true effect because subjects are randomly assigned to groups, 5% of tests should report a significant difference when α = 0.05 (Fig 1A). We found, however, that fewer tests than expected reported a significant difference (*P* = 0.005). Many studies have documented a selection bias against nonsignificant results [2,4,11], but, to our knowledge, ours is the first to present evidence of a selection bias for nonsignificant results.

## Why are there so few significant results?

Selection bias can arise because of a file-drawer effect (i.e., studies go unpublished) or selective reporting. Although the former is possible, we suspect that researchers would still attempt to publish studies even if a confounding variable differed significantly between the control and treatment group. Reviewers might, however, occasionally block publication on the grounds that the allocation of subjects means that causal inferences about a manipulated variable are no longer possible (although one could, of course, statistically control for the confounding variable). We therefore focus on selective reporting as the main reason for a dearth of significant results. While selectively reporting goes against recent calls for research transparency (for example, [20]), the failure to report the outcome of a test need not mean that investigators purposefully omitted results that undermine a study’s validity. Based on reading the Methods of many articles, it is clear that some studies did not report a test for a difference between treatment(s) and control groups in a confounding variable despite the data existing (i.e., confounding variables were measured). We estimate there were up to 371 such studies, but we note that this is a subjective claim, and we may have incorrectly classified some articles. A lack of test results could have occurred because the relevant statistical test was not performed or because the test output was not reported. It is impossible to differentiate between these possibilities, unless there is selective reporting such that missing *P*-values are unevenly distributed between 0 and 1.

The fact we observed too few studies with *P*-values less than 0.05 implicates the selective reporting of nonsignificant results. The magnitude of this type of bias could, however, be underestimated (and might sometimes even go undetected) when examining *P*-curves if there is additional under-reporting of precise, nonsignificant *P*-values. Omission of nonsignificant results might stem from a perception that they are uninteresting [2, 4, 6, 21]. Indeed, some studies in our dataset tested up to 17 confounding variables and then provided statements like ‘all NS’ or ‘all *P* > X’ rather than precise *P*-values. These results had to be excluded from the *P*-curve, which required that *P*-values were stated to two decimal places. The net effect of the inclusion of results reported as ‘*P* > 0.05’ or ‘NS’ would be to increase the frequency of studies with *P*-values above 0.05. In addition, in ‘*P* greater than’ statements, the most frequent *P*-value used was 0.05. This might further explain why we observed so few exact *P*-values in the 0.05 to < 0.10 bin (Fig 1C). Researchers might prefer not to provide exact *P*-values when they lie close to 0.05 (i.e., below 0.10) because such findings are often described, albeit erroneously, as ‘marginally significant’. Crucially, however, if all nonsignificant results are under-reported, as occurs with conventional selective reporting, the *P*-curve would reveal an excess of significant *P*-values. We found the opposite. There is high incentive to publish completed experiments, and we suggest that not reporting significant tests for a confounding variable is the least laborious way to handle such an unwelcome result.

Reverse *P*-hacking is another reason for fewer than expected significant results, even if all *P*-values that are calculated are then published. It is, however, a challenge to determine whether it is occurring (see responses to [9]). Our approach to test for its existence can be illustrated by considering the complementary phenomenon of *P*-hacking when researchers are rewarded for obtaining significant results. Theory states that a *P*-curve is flat if there is no true effect and right skewed if there is a nonzero true effect (i.e., small *P*-values are more common). One can test for *P*-hacking by only looking at significant *P*-values because within this portion of the *P*-curve, there should be no selection bias. One can then test if the curve here is left skewed, with fewer highly significant than barely significant results, indicative of *P*-hacking [10,22]. This would, however, require extreme levels of *P*-hacking if there is actually a nonzero true effect. As such, it has been suggested that one can simply test if there are more *P*-values immediately below 0.05 than further away (for example, 0.045 to < 0.050 versus 0.040 to < 0.045) as evidence for *P*-hacking (for example, [9]; but see [12]).

Here, we pursued a similar finer-scale *P*-curve analysis to test for reverse *P*-hacking. We compared the frequency of *P*-values in two equal-sized bins immediately above the 0.05 threshold (Fig 1B and 1C). We predicted that there would be more in the 0.05 to < 0.10 bin than the 0.10 to < 0.15 bin. This assumes that an initially significant *P*-value is incrementally shifted toward a larger value as, for example, individual data points are selectively added or removed from a large dataset. If a researcher stops reverse *P*-hacking once the observed *P*-value becomes nonsignificant, it is then likely to fall into the 0.05 to < 0.10 bin. This prediction was not supported. There were actually fewer *P*-values in the 0.05 to < 0.10 than 0.10 to < 0.15 bins (Fig 1C). In hindsight, we suggest that many forms of reverse *P*-hacking will shift initially significant values well beyond the 0.05 to < 0.10 bin, and the exact distribution of reverse *P*-hacked values is thus hard to predict. A comparable reservation has been made about the distribution of *P*-hacked values (i.e., that they will not preferentially lie immediately below 0.05; [12]). It is intriguing to note, however, that we also documented too few values in the 0.05 to < 0.10 bin (Fig 1C). These values are often, albeit incorrectly, described as ‘marginally significant’, raising the possibility that they are disliked by researchers wanting to present an image of their experiment as having eliminated differences in confounding variables between control and treatment groups. Selection bias and/or reverse *P*-hacking could therefore explain the general lack of *P*-values below 0.10.

## Summary and conclusions

Although there was a strong signal of missing *P*-values below 0.10, we cannot determine whether this is due to selective reporting, reverse *P*-hacking, or both. The most parsimonious explanation is selective reporting, but we cannot exclude reverse *P*-hacking. We collected data from behavioural ecology articles based on our own expertise in this discipline, but there is no a priori reason to believe that behavioural ecologists treat confounding variables any differently than researchers in other disciplines (for example, [16,17]). Regardless of the proximate mechanism, we have provided further evidence by taking a novel, to our knowledge, approach that poor research practices arise from an obsession with *P*-values and discriminating between ‘significant’ and ‘nonsignificant’ findings (see also [2,4,6,9,11,22]). A focus on *P*-values rather than effect sizes is clearly problematic. We encourage others to test for reverse *P*-hacking and/or a selection bias against nonsignificant results in their own research fields in equivalent tests of confounding variables. This will allow us to assess the generality of the pattern we observed. Finally, it should be noted that the interpretation of recent studies is unlikely to be affected by group differences in confounding variables. Earlier studies often used univariate tests (for example, *t* tests or Mann–Whitney U tests) that ignored major confounding variables (i.e., implicitly assumed that they did not differ between the control and treatments group). In contrast, recent studies usually add potential confounding variables as covariates that are ‘corrected for’ before examining the effect of an experimental intervention.

## Supporting information

S1 Text Contains details of the preregistration of the study, links to data files and an R script, and details of the protocol for data collection and analysis. (DOCX)
