# Supplementary material for: Evidence that nonsignificant results are sometimes preferred: Reverse P-hacking or selective reporting?
Source: PLoS Biol. 2019 Jan 25;17(1):e3000127. doi: 10.1371/journal.pbio.3000127 (PMC6364929; doi:10.1371/journal.pbio.3000127)
Supplement: S1 Text — (DOCX) [file pbio.3000127.s001.docx]

**S1_Text**

**Supporting Information**

***Study pre-registration***

To reduce our own risk of data manipulation we pre-registered our study design prior to data collection through the Center for Open Science online platform (registration form link: <https://osf.io/xg6ja/>). Subsequent modifications to the original design are explicitly noted below. The data file can be accessed at either <https://figshare.com/s/f3bb7dfefdaa8976d3a1> or <https://osf.io/au7yc/>.

***Data collection***

On 1 February 2018, we searched for articles published between 1990 and 2018 in three leading behavioural ecology journals: *Animal Behaviour, Behavioral Ecology*, and *Behavioral Ecology and Sociobiology,* that had ‘experiment* AND control*’ in the ‘topic’ category in the ISI Web of Science database. These journals were chosen as the contain studies in our field of expertise. We are therefore familiar from our own primary work with the various study designs, and ways of presenting results. Our own expertise facilitated data collection as the papers often needed to be read closely, which is a laborious task. All three journals are considered to be prestigious venues for publication by behavioral ecologists, have comparable impact factor and are generally considered by those in the field to be a desirable place to publish (*Animal Behaviour* and *Behavioral Ecology* are the society journals for the two leading academic societies that study the behavior and behavioural ecology of non-domesticated animals). The period 1990-2018 was used because we wished to sample the journals as evenly as possible and *Behavioral Ecology* only started publishing in 1990.

We downloaded 1081 papers that matched these criteria and then created an alphabetical list based on their titles. We then sequentially assigned papers between two of us (MV and PC) to ensure that the order of data collection was random with regard to year of publication and the first author’s surname. We then searched these articles for tests of whether the mean value of confounding variables differed between treatment(s) and control groups where subjects were randomly assigned to groups. We only included confounding variables that involved measurements made on test subjects or groups of subjects (e.g. body mass, blood glucose, brood size) as those variables are more likely to be actively randomized between treatment groups than abiotic variables (e.g. temperature, photoperiod).

To identify papers with suitable data, we first read the Abstract to see if there was any indication that the study was likely to include tests for confounding variables differing between control and treatment groups (i.e. studies that involved experimental manipulation). If the Abstract suggested the paper might be appropriate, we further inspected the Methods section for relevant statistical tests. We looked for the associated P-value or a written statement that the test yielded a significant or non-significant finding. When we could not find the outcome of the test in the Methods, we also checked the Results section. We recorded: the name of the confounding variable, P-values or statistical significance statements associated with tests for a difference, the sample sizes, and what type of test was used (e.g. *t*-test, ANOVA, Mann-Whitney U-test).

In our pre-registration methodology we proposed a sample size of 500 articles with usable data. However, suitable articles were far rarer than expected. On 9 May 2018, we therefore extended the set of papers we examined by expanding the original search criteria to include ‘experiment* NOT control*’. This yielded an additional 3756 articles that we processed in the same way as the original set. The low frequency of suitable articles despite this extended search led to our decision to reduce the final sample size to 250 articles. We amended our online study registration when we reached 220 suitable articles. Our new stopping rule was 250 articles. We did not analyse our results prior to the completion of data collection. We performed cross-validation between the two investigators that collected data once the 250 articles had been processed. We randomly sampled 25 papers examined by each investigator and assigned them to the other investigator to assess the article’s suitability and to retrieve any usable data. Cross-validation led to 94% concordance (i.e. discordance on suitability for 3 of 50 articles).

***Data analysis***

We tested the null hypothesis that 5% of articles that test for a difference in the mean value of a confounding variable between treatment(s) and control will report a significant P-value (i.e. P <0.05) based on an even distribution of P-values (i.e. flat ‘P-curve’) given a true effect size of zero (Fig 1a). We used a one-tailed exact binomial test to compare the number of studies with non-significant and significant test outcomes (run using *binom.test* in R 3.5.0 (R Development Core Team)). The test is one-tailed because of the strong prior prediction that significant results are disfavoured (see Introduction). The majority of the usable articles (60%, 150/250) reported tests for more than one confounding variable. To avoid pseudo-replication, and to take a highly statistically conservative approach, each article only contributed a single test to the final dataset; and, if an article contained at least one significant statement of a difference in confounding variables between treatment groups, we recorded it as ‘significant’.

For further exploratory analyses (not described in our OFS pre-registration) to try to disentangle selection bias from reverse P-hacking we examined P-curves (Fig 1b) [1], which we constructed by extracting all P-values presented to two or more decimal places (n=188 articles, 427 P-values). We omitted articles providing only general statements about significance (e.g. ‘NS’, n=19) or ‘P > X’ statements (n=46), or P-values reported to one decimal place (n=9). Of the usable articles, 104 tested for more than one confounding variable. To avoid pseudo-replication in this second analysis based on precise P-values, we randomly sampled one P-value per article. We then placed all P-values into bins that were 0.05 units wide (e.g. 0 to <0.05, 0.05 to <0.10, 0.10 to <0.15). If investigators are reverse P-hacking, we expect relatively more P-values in the bin closest to the significance threshold (i.e. between 0.05 and <0.10) than in the next bin (i.e. between 0.10 and <0.15) (Fig 1b) [2] ; but see [3]). To test for a difference in the number of studies in these two bins we ran a one-tailed, exact binomial test. We iteratively performed the test; in each iteration we randomly drew one P-value per article. Here we present the median P-value from the 500 one-tailed, exact binomial tests.

To test for an effect of year of publication we conducted a two-sample *t*-test (one-tailed). This produces identical results to the use of a binary logistic regression of article type (significant/non-significant result of a test for a confounding variable) on year of publication (as described in our OFS pre-registration), but with the advantage that we can additionally report the power to detect a medium strength effect (Hedge’s *d* = 0.5) [4].

**References**

1. Simonsohn U, Nelson LD, Simmons JP. P-Curve: A key to the file-drawer. J Exp Psychol-Gen. 2014;143: 534-547. doi: 10.1037/a0033242 PMID: 23855496

2. Head ML, Holman L, Lanfear R, Kahn AT, Jennions MD. The extent and consequences of p-hacking in science. PLoS Biol. 2015;13: e1002106. doi: 10.1371/journal.pbio.1002106 PMID: 25768323

3. Bishop DVM, Thompson PA. Problems in using *p*-curve analysis and text-mining to detect rate of *p*-hacking and evidential value. PeerJ 2016;4: e1715. doi: 10.7717/peerj.1715 PMID: 26925335

4. Cohen J. Statistical Power Analysis for the Behavioral Sciences. 2^nd^ ed. New Jersey: Lawrence Erlbaum, 1988.
